# Can Robots Encourage Social Engagement Among Older Adults?

**DOI:** 10.1093/geroni/igaa057.624

**Published:** 2020-12-16

**Authors:** Yichun Lin, Jing Fan, Mary Dietrich, Linda Beuscher, Paul Newhouse, Nilanjan Sarkar, Lorraine Mion

**Affiliations:** 1 the Ohio State University, Columbus, Ohio, United States; 2 Vanderbilt University, Nashville, Tennessee, United States; 3 The Ohio State University, Columbus, Ohio, United States

## Abstract

Apathy in older adults in long term care (LTC) settings is common, associated with morbidity and mortality, and requires extensive personnel time. Most LTC sites are limited in their ability to provide activities. We conducted a 3-month pilot study at two LTCs to determine whether a robot, with/without virtual reality (VR), was successful in encouraging social engagement between older LTC adults. Three robot activities were offered twice weekly for three weeks (6 sessions). Two activities with VR consisted of two book sorting games. One activity was “Simon Says” where older adults took turns as leaders. Demographics and cognitive data were collected. Videos were coded and analyzed using Noldus Observer: activity engagement as visual and verbal attention to the robot activity and social engagement as visual and verbal attention towards their partner. Participants were 2 men and 14 women, mean age 83. One dropped out because of hearing problems; one dropped out because of cognitive impairment. Fourteen, ie 7 pairs, attended all 6 sessions; ten had MCI and one had AD. Social and activity engagement varied by activity and by participant. Participants’ perceptions (7-point Likert scale) remained positive over time (6.33 (SD 0.94) to 6.52 (SD 0.61)) but decreased slightly for the repeat activities (6.19 (SD 1.01) to 5.96 (SD 1.13)). Robots hold promise in LTC as ways to engage older adults who suffer from apathy. Further work is necessary to elucidate participant- and activity-level characteristics most conducive for success and mechanisms to increase the number and variety of activities.

